# Isolation and Genetic Characterization of Parvoviruses From Dogs, Cats, Minks, and Raccoon Dogs in the Eastern Region of Shandong Province, China

**DOI:** 10.3389/fmicb.2022.862352

**Published:** 2022-02-28

**Authors:** Jiakai Zhao, Hao Zhang, Lu Zhang, Qiang Zhang, Ning Zhou, Taofeng Du, Qin Zhao, En-Min Zhou, Yongkun Du, Yani Sun

**Affiliations:** ^1^Department of Preventive Veterinary Medicine, College of Veterinary Medicine, Northwest Agriculture and Forestry University, Yangling, China; ^2^Scientific Observing and Experimental Station of Veterinary Pharmacology and Diagnostic Technology, Ministry of Agriculture, Yangling, China; ^3^College of Veterinary Medicine, Henan Agricultural University, Zhengzhou, China

**Keywords:** canine parvovirus 2, feline panleukopenia virus, genetic characterization, mink enteritis virus, raccoon dog parvovirus, phylogenetic analysis

## Abstract

The eastern region of Shandong province, China, is an intensive economic mink and raccoon dog breeding area. To investigate the molecular variations of parvovirus in cats, dogs, minks, and raccoon dogs from this region, feline panleukopenia virus (FPV), canine parvovirus 2 (CPV-2), mink enteritis virus (MEV), and raccoon dog parvovirus (RDPV) were separately isolated and characterized from the respective animals with gastroenteritis. PCR amplification showed that there were 15/18 (83.3%), 9/13 (69.2%), 8/11 (72.7%), and 3/7 (42.9%) samples from the diseased animals separately positive for FPV, CPV-2, MEV, and RDPV. Of these, a total of six FPV, six MEV, four CPV-2, and three RDPV strains were successfully isolated using F81 cells. Next, the near-complete genomes of 19 parvovirus isolates were amplified and analyzed. The viral particle 2 (VP2) sequence alignment showed that they shared 97.2–100% nucleotide similarity. Phylogenetic analysis showed that the five FPV isolates were in the same branch, and an FPV isolate was closely related with MEV and RDPV isolates obtained in this study. These suggested that cross-species infection occurred in the Shandong region between the FPV, MEV, and RDPV. For the four CPV-2 isolates, three were antigenic variant strains CPV-2a, and the other was antigenic variant strain CPV-2c. Additionally, the mutations that had emerged in the VP2 amino acids of CPV-2 also occurred in the VP2 from the FPV, MEV, and RDPV isolates. This study suggested that the continuous evolution of the parvovirus may be accelerated in areas with a high density of economic animal trading/breeding, and controlling parvovirus infection in these animals remains a challenge.

## Introduction

*Parvoviridae* is divided into the three subfamilies: *Parvovirinae*, *Densovirinae,* and *Hamaparparvovirinae*, and these are distinguished primarily by their respective ability to infect vertebrate and arthropod hosts.^1^ Feline panleukopenia virus (FPV), canine parvovirus 2 (CPV-2), mink enteritis virus (MEV), and raccoon dog parvovirus (RDPV) belong to the species *Carnivore protoparvovirus 1* (genus *Protoparvovirus*, family *Parvovirinae*; Cotmore et al., 2019). They are all autonomous, linear, single-stranded, and negative-sense DNA viruses with a genome of approximately 5 kb (Johnson et al., 1974; Ohshima and Mochizuki, 2009). The four viruses are very closely related, showing more than 98% genome similarity (Battilani et al., 2011; Cotmore et al., 2014, 2019). The complete viral genome contains two large open reading frames encoding for two structural (VP1 and VP2) and two non-structural (NS1 and NS2) proteins (Reed et al., 1988). The VP2 protein contains the primary antigenic epitopes of viral particles and plays an important role in viral pathogenicity and hosts ranges (Truyen, 2006).

Feline panleukopenia, a disease of cats caused by FPV, was initially reported in the 1920s and can infect many species within the *Carnivora* order, including cats, dogs, minks, raccoon dogs, and foxes (Steinel et al., 2000). As a highly contagious sickness occurring in cats, FPV infection causes severe leukopenia and gastroenteritis (Steinel et al., 2001; Muz et al., 2012). In 1952, MEV, the causative pathogen of viral enteritis in minks, was characterized (Wills, 1952; Yuan et al., 2014). In the 1970s, it was documented that MEV was serologically and biochemically related to FPV and a variant strain of FPV (Parrish, 1990). As a variant of feline parvovirus, CPV-2 was first recognized in the mid-1970s and was shown to cause severe fatal epizootics of gastroenteritis in dogs (Appel et al., 1979; Truyen, 1999; Hoelzer and Parrish, 2010). Three variants of CPV-2, CPV-2a, CPV-2b, and CPV-2c have been identified worldwide (Li et al., 2019). It also had a broad host range and naturally infected cats (*Felis catus*), raccoons (*Procyon lotor*), and raccoon dogs (*Nyctereutes procyonoides*; Allison et al., 2012). RDPV was first isolated from raccoon dogs in China in 2018 and is known as a variant of CPV-2 (Jia-Yu et al., 2018). Once the raccoon dog is infected with parvovirus, it experiences severe enteritis, including vomiting, hemorrhagic diarrhea, depression, loss of appetite, and dehydration (Jia-Yu et al., 2018). The infections caused by these four viruses seriously affect the health of pets and the development of particular economic animal industries in China (Barker et al., 1983; Steinel et al., 2001). In addition, it was documented that the CPV-2 can infect species among dogs, cats, minks, and raccoon dogs, and FPV can infect species among cats and minks, particularly in areas where special economic animals are intensively raised, which presents a considerable challenge for the prevention and control of the four diseases (Truyen et al., 1995; Lu et al., 2020).

The eastern region of Shandong province in China is an intensive area of economic mink and raccoon dog breeding. Between 2018 and 2019, the incidence of gastroenteritis in dogs, cats, minks, and raccoon dogs in this area increased, resulting in major economic consequences. In the present study, the fecal samples from the diseased cats and dogs and intestinal samples from the minks and raccoon dogs from this area were collected and used to isolate parvovirus. Next, the genetic characterizations of these isolated parvoviruses were analyzed.

## Materials and Methods

### Ethics Statement

Based on the Guide for the Care and Use of Laboratory Animals of the Northwest Agriculture and Forestry University (NWAFU) recommendations, the clinical samples were collected for processing. The protocols were approved by the IACUC of NWAFU (approval no. 20180014/02).

### Cells and Samples

F81 cells were purchased from American Type Culture Collection, cultured in RPMI 1640 (Biological Industries) supplemented with 10% fetal bovine serum (FBS; Gibco; Thermo Fisher Scientific, Inc.), in a humidified incubator at 37°C with 5% CO_2_.

Between 2018 and 2019, 18 fecal samples from cats and 13 samples from dogs were collected from the Veterinary Hospital in the eastern region of Shandong province, China. The cats and dogs exhibited diarrhea, emesis, and dehydration, which approximately accounts for 11.5% of the hospital cases. In addition, the 11 minks from which intestinal samples were obtained and the seven raccoon dogs also exhibited diarrhea and were collected from seven farms with the amount of livestock being 1860 minks and 395 raccoon dogs also located in the Shandong province; the Veterinary Hospital is located near these farms. All samples were first emulsified in 1 ml 0.1 M phosphate-buffered saline (pH 7.4) and centrifuged at 3,500 *g* for 10 min at 4°C. Next, the supernatants were collected and used for DNA extraction and virus isolation.

### Detection of Viral DNA From the Samples

First, universal primer pairs were designed for detecting the viral DNA of FPV, CPV-2, MEV, and RDPV. Based on the highly conserved VP2 gene of the four viral genomes, a primer pair (VP2-S1 and VP2-S2) was designed and synthesized (Table 1). The expected size of amplification with the primers was approximately 571 base pairs (bp). Second, the total viral DNA was extracted from 200 μl of the supernatants mentioned above from the samples after processing using the EasyPure Viral DNA/RNA kit (TransGen Biotech, CO., Ltd.) to the manufacturer’s instructions. Briefly, the unique lysate was used to cleave the virus and release DNA, which was adsorbed by a silica gel-membrane centrifuge column (TransGen Biotech, CO., Ltd.) and centrifuged with 10,000 *g* for 2 min at 4°C to purify viral DNA effectively. The target DNA fragments were amplified by PCR using the primer pairs VP2-S1 and VP2-S2. The PCR reaction consisted of 1 U TransTaq^®^ HiFi DNA Polymerase (TransGen Biotech, Co., Ltd.), 5 μl TransTaq^®^ HiFi Buffer 10 X (TransGen Biotech, Co., Ltd.), 10 mM each dNTP (4 μl, TransGen Biotech, Co., Ltd.), 100 ng viral DNA, and 10 μM of each primer (1 μl). The amplification conditions were as follows: Initial denaturation at 94°C for 5 min; followed by 34 cycles of denaturation at 94°C for 30 s, annealing at 55°C for 35 s and extension at 72°C for 35 s; and with a final extension of 72°C for 5 min. The PCR products were analyzed by electrophoresis using a 1% agarose gel and sequenced by TINGKE Biological Technology Company. The obtained sequences were analyzed for genetic identification by performing BLAST searches.^2^

**Table 1:** Primers used in this study.

Primers	Sequences (5′–3′)	Size (bp)
VP2-S1	GGATTTCTACGGGTACTTTC	571
VP2-S2	GGTGTGCCACTAGTTCCAGTAT	
P1	GTCTGGCAACCAGTATACTGAGG	2,400
P2	GATGTTGATGGATGATCTGG	
P3	TCTCGCCAGCAGATCAACGCTT	2,026
P4	CATAAAAACATAGTAAGTATG	

### Viral Isolation

To isolate the virus, the supernatants from the clinical samples positive on PCR detection were filtered through a 0.22-μm Millipore filter (Millipore Sigma) and subsequently inoculated into F81 cells of 2 × 10^7^ cells in a final volume of 100 μl. The inoculated cells were maintained with 3% FBS at 37°C in a humidified 5% CO_2_ incubator and monitored daily for the cytopathic effects (CPEs), including floating, rounding, or disintegration. When a CPE was observed in 80% of the inoculated cells, the cells were collected and freeze-thawed three times. After centrifugation with 8,000 *g* for 10 min at 4°C, the virus’s supernatants were collected and stored −80°C. If no CPE was observed in the inoculated cells, the supernatants were further inoculated until a CPE was observed. All supernatants were added to F81 cells for five passages. Next, an indirect immunofluorescence assay (IFA) was performed on the F81 cells inoculated with the five-passaged supernatants for confirming viral isolation. In addition, the five-passaged supernatants were also assessed for viral DNA by PCR as described above.

### Immunofluorescence Assay

To confirm that the virus had been successfully isolated and had infected the cells, IFA was performed. Briefly, after the F81 cells in the six-well plates were inoculated and cultured with the supernatants containing the virus for 2 days, they were fixed for 30 min at 4°C with pre-cooled 70% ethanol. Then, the cells were incubated in 1% BSA for 1 h at 37°C to block non-specific protein–protein interactions. Subsequently, the cells were incubated with mouse anti-CPV-2 monoclonal antibodies cross-reacting with FPV, MEV, and RDPV (Qianxun Biological Company, catalog. no: Ab-015) using the dilution of 1:50 overnight at 4°C. The secondary antibody used was a FITC-goat anti-mouse IgG antibody (Jackson ImmunoResearch Laboratories, Inc., catalog. no: 115–035\u2013003) with a dilution of 1:200 and incubated for 1 h at 37°C. Finally, a fluorescent strain (4′,6-diamidino-2-phenylindole, DAPI) for 5 min at room temperature was used to stain the cell nucleus. The cells were observed under a fluorescence microscope with a 10 × objective (Leica AF6000, Germany).

### Amplification of the Near-Complete Genome of the Four Viruses

To amplify the near-complete genome of FPV, CPV-2, MEV, and RDPV isolated in the present study, the two primer pairs (P1, P2, P3, and P4) were designed based on the reference sequences in GenBank (accession no. MH106700). The sequences of the primers are shown in Table 1. The expected product size was 2,400 bp with the primers P1 and P2 and 2,026 bp with the primers P3 and P4. PCR was performed as described above for detecting the viral DNA from the five-passaged supernatants, with the only difference being the viral template DNA. The conditions for PCR amplification were also the same as that described above, except that the 35 s of extension time was extended to 1 min. The positive PCR products were purified using an *EasyPure*^®^ PCR Purification kit (TransGen Biotech, Co., Ltd.) and then cloned into a pMD-19-T vector (Takara Bio, Inc.) based on the manufacturer’s protocol. The plasmids were transformed into competent *Escherichia coli* DH5α cells. For sequencing, the positive clones were sent to Sangon Biotech, Co., Ltd. The near-complete genomic sequences were edited and assembled using the EditSeq program 7.1.0 of the Lasergene software package (DNASTAR, Madison, WI, United States). All the sequences were determined from at least three independent PCR products.

### Sequences and Phylogenetic Analysis

Based on the near-complete genome, the phylogenetic tree was constructed using the sequences obtained in the present study and partially referenced sequences from GenBank using the neighbor-joining method in MEGA 6.0 software. The GenBank accession nos. of the FPV, CPV-2, MEV, and RDPV sequences are shown in Table 2. The robustness of the tree was assessed by bootstrap analyses using 1,000 re-samplings of the data.

**Table 2:** Nucleotide and amino acid homology of the isolates.

Isolates	Identity (%)																			
	Accession number	1	2	3	4	5	6	7	8	9	10	11	12	13	14	15	16	17	18	19
**CPV-SD/2019/1**	**OK384305**		100	99.8	99.3	99.3	98.3	98.3	98.3	98.3	98.3	98.5	99.0	99.1	99.1	99.1	99.1	99.1	99.0	99.0
**CPV-SD/2019/2**	**OK384306**	100		99.8	99.3	99.3	98.3	98.3	98.3	98.3	98.3	98.5	99.0	99.1	99.1	99.1	99.1	99.1	99.0	99.0
**CPV-SD/2019/3**	**OK384307**	100	100		99.0	99.1	98.1	98.1	98.1	98.1	98.1	98.3	98.9	99.0	99.0	99.0	99.0	98.9	98.9	98.9
**CPV-SD/2019/4**	**OK384308**	99.3	99.3	99.3		98.7	98.0	98.0	98.0	98.0	98.0	97.9	98.5	98.6	98.6	98.6	98.6	98.7	98.5	98.5
**FPV-SD/2019/1**	**OK384309**	99.1	99.1	99.1	98.8		98.0	98.0	98.0	98.0	98.0	99.1	99.7	99.8	99.8	99.8	99.8	98.8	99.6	99.6
**FPV-SD/2019/2**	**OK384310**	97.4	97.4	97.4	97.1	97.6		100	100	100	100	97.2	97.7	97.9	97.8	97.9	97.9	98.6	97.7	97.7
**FPV-SD/2019/3**	**OK384311**	97.4	97.4	97.4	97.1	97.6	100		100	100	100	97.2	97.7	97.9	97.8	97.9	97.9	98.6	97.7	97.7
**FPV-SD/2019/4**	**OK384312**	97.4	97.4	97.4	97.1	97.6	100	100		100	100	97.2	97.7	97.9	97.8	97.9	97.9	98.6	97.7	97.7
**FPV-SD/2019/5**	**OK384313**	97.4	97.4	97.4	97.1	97.6	100	100	100		100	97.2	97.7	97.9	97.8	97.9	97.9	98.6	97.7	97.7
**FPV-SD/2019/6**	**OK384314**	97.4	97.4	97.4	97.1	97.6	100	100	100	100		97.2	97.7	97.9	97.8	97.9	97.9	98.6	97.7	97.7
**MEV-SD/2019/1**	**OK384315**	97.4	97.4	97.4	97.1	98.3	95.9	95.9	95.9	95.9	95.9		99.1	99.3	99.3	99.3	99.3	98.0	99.1	99.1
**MEV-SD/2019/2**	**OK384316**	98.8	98.8	98.8	98.5	99.7	97.3	97.3	97.3	97.3	97.3	98.3		99.8	99.9	99.8	99.8	98.5	99.7	99.7
**MEV-SD/2019/3**	**OK384317**	99.0	99.0	99.0	98.6	99.8	97.4	97.4	97.4	97.4	97.4	98.5	99.8		99.9	100	100	98.7	99.8	99.8
**MEV-SD/2019/4**	**OK384318**	99.0	99.0	99.0	98.6	99.8	97.4	97.4	97.4	97.4	97.4	98.5	99.8	100		99.9	99.9	98.6	99.8	99.8
**MEV-SD/2019/5**	**OK384319**	99.0	99.0	99.0	98.6	99.8	97.4	97.4	97.4	97.4	97.4	98.5	99.8	100	100		100	98.7	99.8	99.8
**MEV-SD/2019/6**	**OK384320**	99.0	99.0	99.0	98.6	99.8	97.4	97.4	97.4	97.4	97.4	98.5	99.8	100	100	100		98.7	99.8	99.8
**RDPV-SD/2019/1**	**OK384321**	98.5	98.5	98.5	98.1	98.5	97.9	97.9	97.9	97.9	97.9	96.8	98.1	98.3	98.3	98.3	98.3		98.6	98.6
**RDPV-SD/2019/2**	**OK384322**	99.0	99.0	99.0	98.6	99.8	97.4	97.4	97.4	97.4	97.4	98.5	99.8	100	100	100	100	98.3		100
**RDPV-SD/2019/3**	**OK384323**	99.0	99.0	99.0	98.6	99.8	97.4	97.4	97.4	97.4	97.4	98.5	99.8	100	100	100	100	98.3	100	

*VP2 nucleotide sequence homology is shown in the gray background and VP2 amino acid homology is shown in white background*.

**Figure 1:**
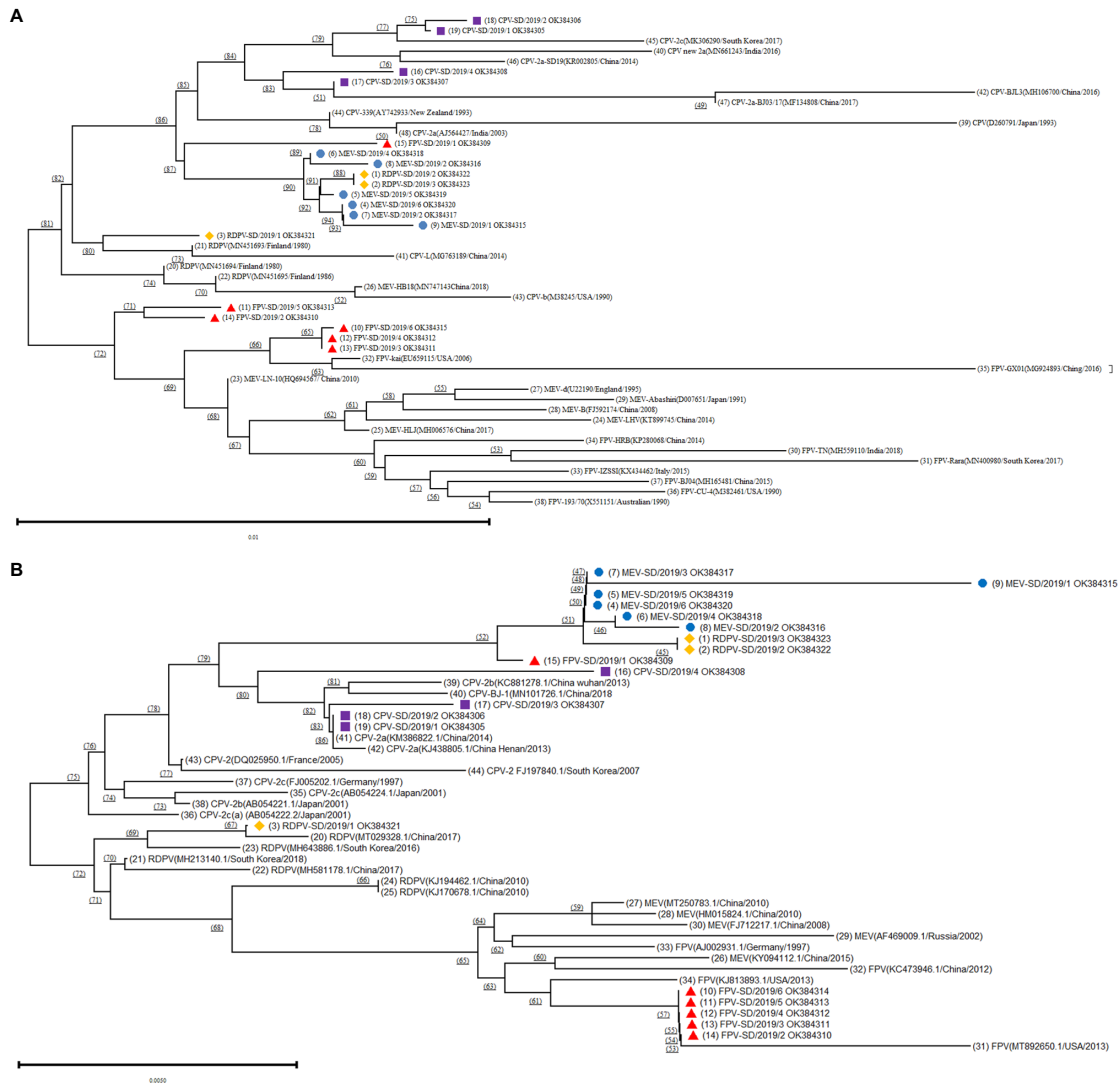
Phylogenetic tree analysis based on the different FPV, canine parvovirus-2 (CPV-2), MEV, and RDPV isolates in the present study and reference isolates from GenBank. **(A)** Construction of the phylogenetic tree based on the near-complete genomes of the different isolates. **(B)** Construction of the phylogenetic tree based on the sequence of the VP2 gene of the different isolates. ■ Represents the CPVs isolated in this study; ▲ represents the FPVs isolated in this study; ● represents the MEVs isolated in this study; and ♦ represents the RDPVs isolated in this study.

In addition, the complete VP2 gene sequences were also obtained by editing from the near-complete genome. Next, the amino acid sequences of the VP2 protein were determined by the translation of the VP2 gene using the EditSeq program. The obtained amino acid sequences of the VP2 protein from the present study and GenBank were aligned using the MegAlign program 7.1.0 of the Lasergene software package (DNASTAR, Madison, WI). Furthermore, the phylogenetic tree based on the VP2 amino acid sequences was constructed using MEGA 6.0 software with 1,000 bootstrap replications (Tamura et al., 2007). The accessions nos. of the sequences downloaded from the GenBank are shown in Figure 1.

## Results

### Genetic Identification of FPV, CPV-2, MEV, and RDPV From the Clinical Samples

A total of 18 clinical fecal samples from the cats were tested for the target gene of FPV by PCR, and the results showed that the expected size (571 bp) bands were successfully amplified in 15 of 18 samples. For the canine samples, it was shown that nine out of 13 samples were positive. In the mink samples, eight out of 11 were positive, and three out of seven were positive in the raccoon dog samples. Next, the 35 positive PCR products were sequenced, and BLAST analysis was performed. The blast results showed that the 15 sequences of the isolates from cats, nine from dogs, eight from minks, and three from raccoon dogs showed high similarity with the VP2 genes of FPV, CPV-2, MEV, and RDPV separately (data not shown).

### Isolation and Identification of FPV, CPV-2, MEV, and RDPV From the Samples

**Figure 2:**
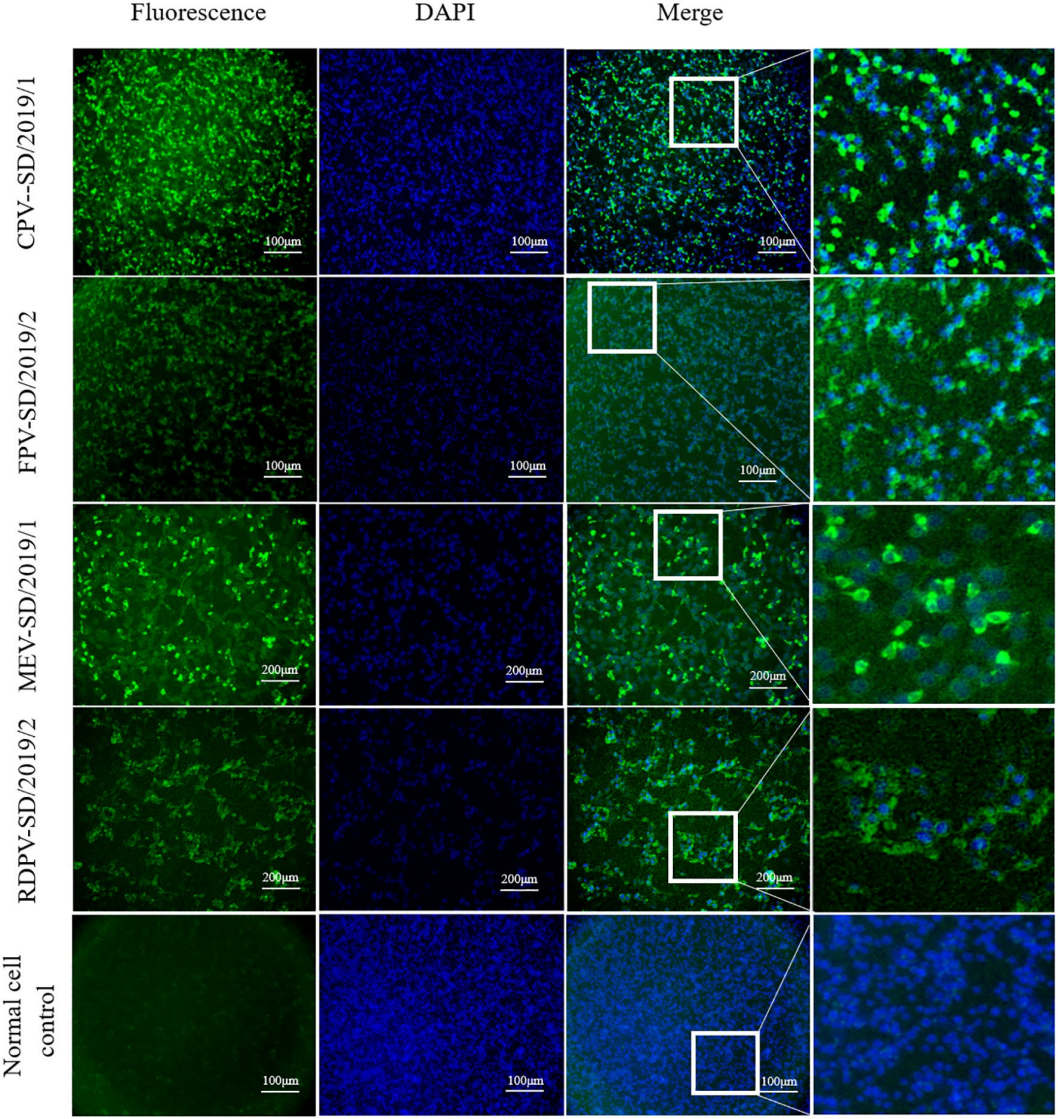
Detection of feline panleukopenia virus (FPV), canine parvovirus (CPV), mink enteritis virus (MEV), and raccoon dog parvovirus (RDPV) in the infected F81 cells by indirect immunofluorescence assay with monoclonal antibodies against the viral particle 2 (VP2) protein of parvovirus.

The positive FPV, CPV-2, MEV, and RDPV samples identified by PCR were inoculated into the F81 cells. At 2 days post-inoculation, the cells inoculated with cat, dog, mink, and raccoon dog viral isolates showed the obvious CPEs, including floating, rounding, disintegration, and increased granularity. The IFA results showed that fluorescence was observed in these inoculated cells, indicating that the viruses in the samples successfully proliferated in the F81 cells (Figure 2). In addition, after five passages in the cells, the supernatants containing the viral stock were still positive for the viral DNA based on PCR amplification. The amplified sequences shared 100% similarity with the respective sequences obtained from the inoculated samples (data not shown). These results indicated that the six FPV, four CPV-2, six MEV, and three RDPV strains were successfully isolated from the clinical feces.

### Amplification and Analysis of the Near-Complete Genomes of FPV, CPV-2, MEV, and RDPV Isolates

The near-complete genomes of six FPV, four CPV-2, six MEV, and three RDPV isolates were amplified using the viral stock from the five-passaged supernatant in the cell as the templates. The sequences of the near-complete genomes were assembled by two overlapping fragment sequences based on at least three independent viral DNA sequences obtained. The near-complete genomes of these isolates were 4,194 nt in length and contained the complete VP1, VP2, and part of the NS1 and NS2 genes. Then, six near-complete genomes of FPV were obtained and termed FPV-SD/2019/1–6. The four CPV-2 sequences were termed CPV-2-SD/2019/1–4. The six MEV genomes were termed MEV-SD/2019/1–6, and the three RDPV genomes were termed RDPV-SD/2019/1–3. All the viral sequences were submitted to GenBank, and the accessions nos. Are presented in Table 2. BLAST analysis showed the nucleotide homologies of the near-complete sequences among the 19 isolates were 98.4–100%, while 97.6–99.9% with the reference strains from the GenBank (Table 3). Out of which, four MEV isolates share low identity (97.6%) with an FPV isolate from South Korea in 2017, CPV-2-SD/2019/3 shares high homology (99.9%) with CPV-2 strain from China in 2018 (Table 3).

**Table 3:** Genome nucleotide homology of the isolates and the reference viruses.

Isolates	Identity (%)																															
	1	2	3	4	5	6	7	8	9	10	11	12	13	14	15	16	17	18	19	20	21	22	23	24	25	26	27	28	29	30	31	32
**CPV-SD/2019/1**		99.9	99.5	99.2	99.0	98.8	98.7	98.6	98.7	98.6	99.0	98.9	99.0	99.0	99.0	99.0	99.1	99.0	99.0	99.5	99.5	99.3	99.4	98.5	98.7	98.0	97.9	98.6	98.7	98.3	98.7	99.3
**CPV-SD/2019/2**			99.4	99.1	99.0	98.7	98.6	98.5	98.6	98.5	98.9	98.9	98.9	99.0	99.0	98.9	99.0	98.9	98.9	99.4	99.4	99.2	99.3	98.5	98.6	97.9	97.8	98.5	98.6	98.1	98.6	99.2
**CPV-SD/2019/3**				99.7	99.1	99.2	98.8	98.7	99.0	98.7	99.4	99.3	99.4	99.4	99.4	99.4	99.4	99.3	99.3	99.9	99.5	99.4	99.0	98.6	98.8	98.2	97.9	98.7	98.8	98.3	98.8	99.4
**CPV-SD/2019/4**					99.9	99.1	98.6	98.6	98.9	98.6	99.1	99.1	99.1	99.2	99.2	99.1	99.2	99.1	99.1	99.7	99.3	99.3	99.3	98.5	98.6	98.0	97.8	98.5	98.6	98.0	98.7	99.3
**FPV-SD/2019/1**						98.7	99.2	99.2	98.5	99.2	99.2	99.2	99.2	99.3	99.2	99.2	99.0	99.2	99.2	99.1	99.3	99.2	98.5	98.7	98.6	98.0	97.9	98.6	98.7	98.0	98.8	99.2
**FPV-SD/2019/2**							99.5	99.5	99.7	99.4	98.9	98.8	98.9	98.9	98.9	98.9	99.2	98.8	98.8	99.2	99.2	99.3	98.4	99.0	99.3	98.6	98.5	99.0	99.1	99.0	99.2	99.3
**FPV-SD/2019/3**								100.0	99.3	100.0	98.5	98.4	98.5	98.5	98.5	98.5	98.9	98.4	98.4	98.7	99.1	99.2	98.3	99.2	99.2	98.6	98.5	99.1	99.2	98.8	99.3	99.2
**FPV-SD/2019/4**									99.3	100.0	98.4	98.4	98.4	98.5	98.5	98.4	98.9	98.4	98.4	98.7	99.1	99.2	98.2	99.2	99.3	98.6	98.5	99.1	99.2	98.9	99.3	99.2
**FPV-SD/2019/5**										99.3	99.2	99.0	99.2	99.1	99.1	99.2	99.0	99.0	99.0	99.0	99.0	99.1	98.3	98.8	99.1	98.5	98.3	98.9	99.1	98.8	99.0	99.2
**FPV-SD/2019/6**											98.4	98.4	98.4	98.5	98.5	98.4	98.9	98.4	98.4	98.7	99.1	99.2	98.2	99.1	99.2	98.6	98.5	99.1	99.2	98.8	99.3	99.2
**MEV-SD/2019/1**												99.8	100.0	99.9	100.0	100.0	99.0	99.9	99.9	99.3	99.2	99.1	98.5	98.3	98.5	97.9	97.6	98.4	98.5	97.9	98.5	99.1
**MEV-SD/2019/2**													99.8	99.9	99.8	99.8	99.0	99.8	99.8	99.3	99.1	99.0	98.4	98.3	98.4	97.8	97.6	98.3	98.5	97.8	98.4	99.0
**MEV-SD/2019/3**														99.9	100.0	99.0	99.0	99.9	99.9	99.3	99.2	99.1	98.5	98.3	98.5	97.9	97.6	98.4	98.5	97.9	98.5	99.1
**MEV-SD/2019/4**															100.0	99.9	99.1	99.9	99.9	99.4	99.3	99.1	98.5	98.4	98.5	97.9	97.7	98.4	98.6	97.9	98.5	99.1
**MEV-SD/2019/5**																100.0	99.1	99.9	99.9	99.4	99.3	99.1	98.5	98.4	98.5	97.9	97.7	98.4	98.6	97.9	98.5	99.1
**MEV-SD/2019/6**																	99.0	99.9	99.9	99.3	99.2	99.1	98.5	98.3	98.5	97.9	97.6	98.4	98.5	97.9	98.5	99.1
**RDPV-SD/2019/1**																		99.0	99.0	99.3	99.4	99.5	98.7	98.9	98.9	98.3	98.1	98.8	98.9	98.6	99.0	99.6
**RDPV-SD/2019/2**																			100.0	99.3	99.2	99.0	98.5	98.3	98.4	97.8	97.6	98.3	98.5	97.9	98.5	99.1
**RDPV-SD/2019/3**																				99.3	99.2	99.0	98.5	98.3	98.4	97.8	97.6	98.3	98.5	97.9	98.5	99.1
**CPV (China, 2018)**																					94.1	91.8	98.9	91.9	95.3	94.4	94.1	98.7	94.3	98.2	90.6	99.4
**CPV-L (China, 2018)**																						97.8	99.0	97.2	98.7	98.1	97.9	99.0	98.0	98.7	97.3	99.7
**CPV (USA, 2018)**																							98.9	93.2	98.1	96.4	96.2	99.1	97.2	98.8	94.0	99.9
**CPV-2c (South Korea, 2018)**																								98.1	98.2	97.6	97.4	99.2	98.2	97.8	98.3	98.9
**FPV (China, 2019)**																									99.1	98.5	98.4	99.2	99.2	99.3	97.8	99.1
**FPV-L (China, 2018)**																										98.7	98.7	99.2	99.2	99.0	99.1	99.2
**FPV (India, 2018)**																											98.5	98.6	98.7	98.0	97.1	98.6
**FPV (South Korea, 2017)**																												98.4	98.4	97.7	96.9	98.4
**MEV (China, 2018)**																													99.5	99.2	99.4	99.1
**MEV (China, 2019)**																														99.2	99.3	99.2
**MEV (England, 1996)**																															99.5	98.8
**MEV (Japan, 2007)**																																99.3
**RDPV (Finland, 1980)**																																

### Alignment of the Amino Acid Sequences of VP2 Proteins From the FPV, CPV-2, MEV, and RDPV Isolates

The PCR products of all the VP2 genes from the isolates were cloned into the pMD19-T vector and sequenced. A comparative analysis of the VP2 nucleotide sequences from these strains was performed with the reference strains from the GenBank. BLAST analysis showed that the nucleotide identity of VP2 gene sequences among the 19 isolates was 97.2–100%, and the amino acid identity was 97.4–100% (Table 2). The nucleotide and amino acid homology among the four CPV-2 strains were separately 99.0–100% and 99.3–100% (Table 2). Among the six isolated FPV strains, amino acid and nucleotide similarity were 97.6–100% and 98.0–100%, respectively (Table 2). For the six MEV strains, amino acid and nucleotide similarities were separately 99.1–100% and 99.8–100% (Table 2). For the three RDPVs, amino acid and nucleotide similarities were separately 98.6–100% and 98.3–100% (Table 2).

The 13 key amino acid sites in the VP2 protein determining the viral antigenicity and host range were also analyzed and classified (Buonavoglia et al., 2001; Govindasamy et al., 2003). For CPV-2, based on the key amino acid mutation at residue 426 (Asp426Glu) in the VP2 protein (Buonavoglia et al., 2001), the results showed that CPV-2-SD/2019/1, CPV-2-SD/2019/2, and CPV-2-SD/2019/3 strains were the same as the antigenic subtype CPV-2a, and the CPV-2-SD/2019/4 strain was the same as CPV-2c (Table 4). Interestingly, one FPV strain (FPV-SD/2019/1) possessed only one amino acid mutation (Gly300Ser) compared with the reference CPV-2a strain (Table 4). The other five strains, FPV-SD/2019/2, FPV-SD/2019/3, FPV-SD/2019/4, FPV-SD/2019/5, and FPV-SD/2019/6, had an amino acid mutation (Leu562Val) when compared with the reference strain (Table 4). The six MEV and two RDPV strains (RDPV-SD/2019/2 and RDPV-SD/2019/3) possessed the same key amino acids but possessed one amino acid mutation (Ala300Ser) compared with the reference CPV-2 strain. The key amino acids of RDPV-SD/2019/1 were the same as the reference CPV-2 strain (Table 4).

**Table 4:** Amino acid sequence variations in the VP2 proteins.

Strains	Accession number	Amino acid residues													Genotype
		80	87	93	101	103	297	300	305	323	426	562	564	568	
**CPV-b**	**PVCPVC**	**R**	**M**	**N**	**I**	**A**	**S**	**A**	**D**	**N**	**N**	**V**	**S**	**G**	**CPV-2**
**CPV-2a**	**TQ686671**	**R**	**L**	**N**	**T**	**A**	**A**	**G**	**Y**	**N**	**N**	**V**	**S**	**G**	**CPV-2a**
**LCPV-V204**	**AB054221**	**R**	**L**	**N**	**T**	**A**	**A**	**G**	**Y**	**N**	**D**	**V**	**S**	**G**	**CPV-2b**
**HRB-A6**	**KT156832**	**R**	**L**	**N**	**T**	**A**	**A**	**D**	**Y**	**N**	**E**	**V**	**S**	**G**	**CPV-2c**
CPV-SD/2019/1	OK384305	R	L	N	T	A	A	G	Y	N	N	V	S	G	CPV-2a
CPV-SD/2019/2	OK384306	R	L	N	T	A	A	G	Y	N	N	V	S	G	CPV-2a
CPV-SD/2019/3	OK384307	R	L	N	T	A	A	G	Y	N	N	V	S	G	CPV-2a
CPV-SD/2019/4	OK384308	R	L	N	T	A	A	G	Y	N	E	V	S	G	CPV-2c
**PLI-IV**	**D88287**	**K**	**M**	**K**	**T**	**V**	**S**	**A**	**D**	**D**	**N**	**L**	**N**	**A**	**FPV**
FPV-SD/2019/1	OK384309	R	L	N	T	A	A	S	Y	N	N	V	S	G	CPV-2
FPV-SD/2019/2	OK384310	K	M	K	T	V	S	A	D	D	N	V	N	A	FPV
FPV-SD/2019/3	OK384311	K	M	K	T	V	S	A	D	D	N	V	N	A	FPV
FPV-SD/2019/4	OK384312	K	M	K	T	V	S	A	D	D	N	V	N	A	FPV
FPV-SD/2019/5	OK384313	K	M	K	T	V	S	A	D	D	N	V	N	A	FPV
FPV-SD/2019/6	OK384314	K	M	K	T	V	S	A	D	D	N	V	N	A	FPV
**MEV-SD1**	**KY094112**	**K**	**M**	**K**	**T**	**V**	**S**	**A**	**D**	**D**	**N**	**V**	**N**	**A**	**MEV**
MEV-SD/2019/1	OK384315	R	L	N	T	A	A	S	Y	N	N	V	S	G	CPV-2
MEV-SD/2019/2	OK384316	R	L	N	T	A	A	S	Y	N	N	V	S	G	CPV-2
MEV-SD/2019/3	OK384317	R	L	N	T	A	A	S	Y	N	N	V	S	G	CPV-2
MEV-SD/2019/4	OK384318	R	L	N	T	A	A	S	Y	N	N	V	S	G	CPV-2
MEV-SD/2019/5	OK384319	R	L	N	T	A	A	S	Y	N	N	V	S	G	CPV-2
MEV-SD/2019/6	OK384320	R	L	N	T	A	A	S	Y	N	N	V	S	G	CPV-2
**RDPV-DP1**	**MF996332**	**R**	**M**	**N**	**I**	**A**	**A**	**A**	**D**	**N**	**N**	**L**	**S**	**G**	**RDPV**
RDPV-SD/2019/1	OK384321	R	M	N	I	A	A	A	D	N	N	L	S	G	CPV-2
RDPV-SD/2019/2	OK384322	R	L	N	T	A	A	S	Y	N	N	V	S	G	CPV-2
RDPV-SD/2019/3	OK384323	R	L	N	T	A	A	S	Y	N	N	V	S	G	CPV-2

*The font in bold is the reference strains, and the normal font is the strains isolated in this study*.

### Phylogenetic Analysis of Different FPV, CPV-2, MEV, and RDPV Isolates

The phylogenetic tree based on the near-complete genomes showed that the CPV-2 isolates in the present study and reference strains from GenBank were present on the same branch (Figure 1A). Interestingly, the one FPV (FPV-SD/2019/1), all six MEV and two RDPV (RDPV-SD/2019/2 and RDPV-SD/2019/3) isolated from the present study were located in the same branch and had a close relationship with CPV-2 isolates (Figure 1A). The results indicated that these isolates might be different variants of the same virus, suggesting that the parvoviruses in circulation in cats, minks, and raccoon dogs were initially derived from the same viral strain. However, the other five FPV isolates (FPV-SD/2019/2–6) and MEV and FPV strains from GenBank were located on the same big branch (Figure 1A). The FPV-SD/2019/3, FPV-SD/2019/4, and FPV-SD/2019/6 isolates showed further FPV-SD/2019/2 and FPV-SD/2019/5 sequences relationships with the reference sequences of MEV and FPV strains (Figure 1A). Another RDPV isolate (RDPV-SD/2019/1) exhibited a close relationship with the reference sequence of the RDPV strain from GenBank (Figure 1A).

The phylogenetic tree based on the VP2 amino acid sequences was similar to that of the near-complete genomes (Figure 1B). It also showed that the FPV-SD/2019/1, RDPV-SD/2019/2, RDPV-SD/2019/3, all six MEV, and four CPV-2 isolates were located on the same big branch (Figure 1B). However, the other five FPV isolates in the study were all on the same branch in the phylogenetic tree constructed based on the VP2 sequences, which was different from the tree based on the genome (Figure 1).

## Discussion

FPV and CPV-2 belong to the genus *Protoparvovirus* in the subfamily *Parvovirinae* and cause serious diseases in cats, dogs, minks, and raccoon dogs (Steinel et al., 2001). These contagious viral diseases are characterized by severe leukopenia, nervous system disorders, and acute hemorrhagic gastroenteritis (Truyen, 2006). Sequence alignments and phylogenetic trees drawn in the present study showed that the four viruses exhibit a high degree of identity and may come from the same ancestral virus. Additionally, it was hypothesized that FPV might be the ancestral virus from which CPV-2, MEV, and RDPV originated for the respective hosts. However, it has been documented that FPV can directly infect minks, and raccoon dogs can be directly infected by CPV-2, indicating that these viruses can cause cross-species infections without adaptive variation. Thus, the four viruses seriously harm the health of pets and the development of the commercial animal breeding industry. In the eastern region of Shandong, the breeding density of minks and raccoon dogs is high, and pet dogs and cats are also raised in these farms. Thus, the epidemiology of the two viruses’ infection in the region was investigated, and FPV and CPV-2 were isolated and characterized from the cats, dogs, minks, and raccoon dogs, in the present study. A total of 19 variants of parvovirus, including six FPV, four CPV-2, six MEV, and three RDPV variants, were isolated and characterized from the four species with enteritis disease in this region. Interestingly, the phylogenetic trees showed one FPV in the middle branches between MEV and RDPV branches, suggesting that there may be a cross-species infection in this region, which also highlights the potential challenge in controlling parvovirus disease in the region.

Feline panleukopenia virus and CPV-2 are two closely related viruses and are widely hypothesized to be variants of FPV infecting a new host (Parrish, 1990). Previous studies have reported that new variants of CPV-2 have acquired the ability to infect felines, allowing them to infect both dogs and cats (Decaro et al., 2010; Clegg et al., 2012). Four mutations in VP2 protein at residues 87, 300, and 305 have been shown to alter the host range in parvoviruses (Stucker et al., 2012). In the present study, we found only one amino acid (Gly300Ser) in the VP2 sequence of FPV-SD/2019/1 isolate that was inconsistent with reference CPV-2a strain and CPV-2a isolates, and other amino acids are consistent, providing novel evidence for the continued emergence of CPV-2 in the feline population in China. We also found only one amino acid (Gly300Ser) in the VP2 sequence of MEV-SD/2019/1–6 isolates inconsistent with CPV-2a isolates, and other amino acids are consistent suggest that CPV-2 cross-infected minks. RDPV was originally derived from CPV-2, suggesting that CPV-2 cross-infected a raccoon dog and mutated during adaptation (Allison et al., 2012). Typically, the genes of RDPV isolates were very similar to that of the original CPV-2, but there were four mutations in the VP2 gene. However, only one amino acid (Gly300Ser) in the VP2 gene of RDPV-SD/2019/1 and RDPV-SD/2019/2 was inconsistent with CPV-2a, and this mutation was consistent with the FPV-SD/2019/1 isolated in the present study. These findings suggested that the cross-species infection of CPV-2a and FPV is more complex than previously suggested, especially given that the isolates were obtained from the mixed breeding region for pets and special economic animals.

To prevent parvovirus infection, these farms of special economic animals in this region were immunized with commercial attenuated vaccines for dogs. However, the outbreak of viral enteritis still occurred on the farms of minks and raccoon dogs, indicating that the commercial dog parvovirus vaccine may not provide complete protection against MEV and RDPV infection in the minks and raccoon dogs. By comparing VP2 amino acids of all isolates in the study, we found that, although the identity between MEV and RDPV and CPV-2 was high, there were still some mutations of key amino acids. These mutations may change the viral antigenicity and cause immunization failure. Thus, molecular epidemiological investigations are necessary to identify the genetic mutation sites of the new viral strains, which is of great significance for revising the immunization plan.

## Conclusion

Collectively, 19 parvovirus variants were successfully isolated from dogs, cats, minks, and raccoon dogs with viral enteritis in the eastern region of Shandong, China. Genetic analysis of these isolates showed that cross-species infections among FPV and CPV-2 occurred in this region’s cats, dogs, minks, and raccoon dogs. Thus, in the areas with a high density of mixed farming in China, the continuous evolution of the parvovirus in different species will likely be accelerated, increasing the difficulty in controlling these animals’ viral spread, thus requiring continuous epidemiological surveillance.

## Data Availability Statement

The datasets presented in this study can be found in online repositories. The names of the repository/repositories and accession number(s) can be found in the article/supplementary material.

## Author Contributions

YS and YD jointly conceived the study. JZ did the experiments and wrote the manuscript. HZ, LZ, and NZ performed the sample collection. QZ and TD contributed to data analyses. QZ, E-MZ, YS, and YD revised the paper. All authors contributed to the article and approved the final manuscript.

## Funding

The study is mainly funded by grants from the National Natural Science Foundation of China (no. 31402233) to QZ and Tang Scholar of Cyrus Tang Foundation to QZ.

## Conflict of Interest

The authors declare that the research was conducted in the absence of any commercial or financial relationships that could be construed as a potential conflict of interest.

## Footnotes

^1^ http://www.ictv.global/report/parvoviridae

^2^ http://www.ncbi.nlm.nih.gov/BLAST
